# The clinical impact of detector choice for beam scanning

**DOI:** 10.1120/jacmp.v15i4.4801

**Published:** 2014-07-08

**Authors:** Jacob A. Gersh, Ryan C. M. Best, Ronald J. Watts

**Affiliations:** ^1^ Gibbs Cancer Center and Research Institute Greer SC; ^2^ Spartanburg Regional Healthcare System Spartanburg SC; ^3^ Department of Radiation Oncology University of Virginia Health System Charlottesville VA; ^4^ Medicine and Radiation Oncology San Antonio TX USA

**Keywords:** commissioning, small fields, modeling

## Abstract

Recently, the developers of Eclipse have recommended the use of ionization chambers for all profile scanning, including for the modeling of VMAT and stereotactic applications. The purpose of this study is to show the clinical impact caused by the choice of detector with respect to its ability to accurately measure dose in the penumbra and tail regions of a scanned profile. Using scan data acquired with several detectors, including an IBA CC13, a PTW 60012, and a Sun Nuclear EDGE Detector, three complete beam models are created, one for each respective detector. Next, using each beam model, dose volumes are retrospectively recalculated from actual anonymous patient plans. These plans include three full‐arc VMAT prostate plans, three left chest wall plans delivered using irregular compensators, two half‐arc VMAT lung plans, three MLC‐collimated static‐field pairs, and two SBRT liver plans. Finally, plans are reweighted to deliver the same number of monitor units, and mean dose‐to‐target volumes and organs at risk are calculated and compared. Penumbra width did not play a role. Dose in the tail region of the profile made the largest difference. By overresponding in the tail region of the profile, the 60012 diode detector scan data affected the beam model in such a way that target doses were reduced by as much as 0.4% (in comparison to CC13 and EDGE data). This overresponse also resulted in an overestimation of dose to peripheral critical structure, whose dose consisted mainly of scatter. This study shows that, for modeling the 6 MV beam of Acuros XB in Eclipse Version 11, the choice to use a CC13 scanning ion chamber or an EDGE Detector was an unimportant choice, providing nearly identical models in the treatment planning system.

PACS number: 87.55.kh

## I. INTRODUCTION

The deviation between measured and actual dose values in small fields are created by a combination of several phenomena: the effects of volume dose averaging resulting from the finite size of the detector, the subsequent perturbation caused by the detector itself, and the disruption of charged particle equilibrium caused by small effective source sizes.^1^, ^2^ While the medical physics community continues to move toward the use of scanning diodes for profile scan acquisition, a recent White Paper by the developers of the Eclipse Treatment Planning System, Varian Medical Systems (Palo Alto, CA), recommend using ionization chambers for all profile measurements.^(3)^ The use of ionization chamber is recommended even for scanning for VMAT and stereotactic applications. The study further suggests that the use of diodes for profile scanning is acceptable, so long as the detector can adequately measure dose in the tail region, suggesting the importance of dose to this region for beam modeling. This study examines the clinical impact of detector choice for profile scanning, with emphasis placed on accuracy in the penumbra and tail regions.

Studies related to dosimetric accuracy have been performed concerning small‐field dosimetry as it relates to the modeling of beams for use in stereotactic applications.^4^, ^5^ While accurate small‐field dosimetry is important for stereotaxy, a broader‐scope manifestation of this phenomenon is demonstrated when scanning beam profiles for beam modeling of treatment planning systems, where accurate dosimetry in the penumbra and the tail is important. Previous studies concerning this specific case clearly show that a broadening of a beam's penumbra can be substantial when comparing measurements using different detectors.^4^, ^5^, ^6^, ^7^, ^8^, ^9^ This effect is also clinically demonstrated by Yan and colleagues.^(10)^ With emphasis placed on the penumbra, methods for reconstructing beam profiles to account for the overestimation of the penumbra are available for ion chambers.^10^, ^11^, ^12^ However, dosimetric modeling accuracy is not attributed solely to the maintenance of penumbral fidelity. Rangel and colleagues^(13)^ demonstrated the influence of dose in the tail of the profile on the beam model, showing that accurate modeling of the penumbra width required accurate measurement in the tail region.

**Figure 1:**
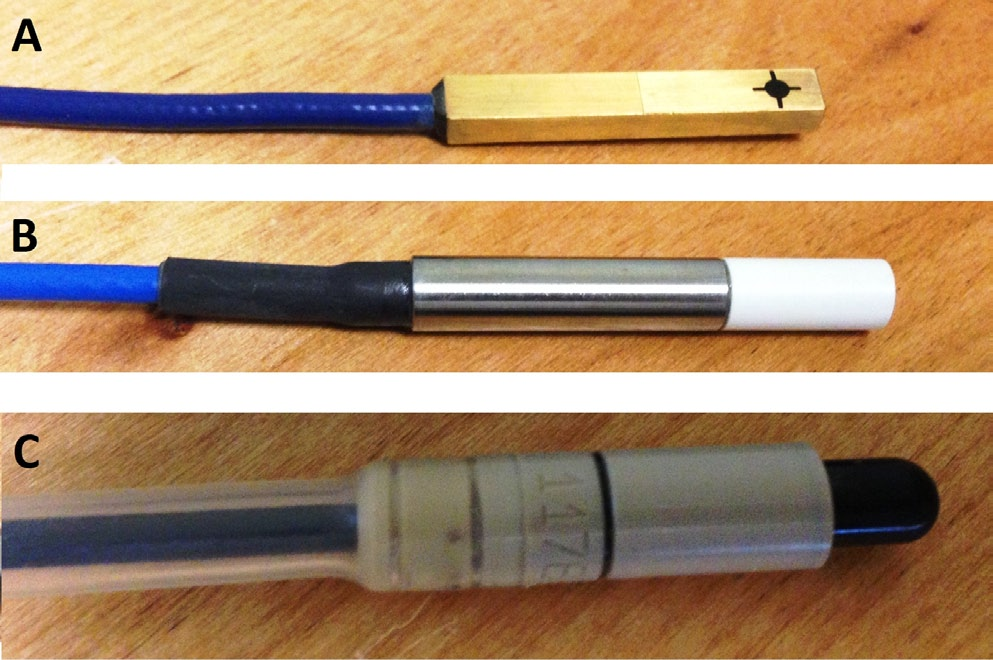
The detectors used in the current study: (a) the Sun Nuclear EDGE Detector scanning diode, (b) the PTW 60012 diode, (c) the IBA CC13 scanning ionization chamber.

In this study, the 6 MV beam of a Varian TrueBeam (Version 1.6; Varian Medical Systems) is modeled in Eclipse (V11) using scan data acquired with several detectors, including a standard scanning ionization chamber, an unshielded scanning diode, and a stereotactic diode detector (shown in Fig. 1). Complete beam models are created for each detector used for scanning. Next, using each beam model, dose volumes are retrospectively recalculated from actual anonymous patient plans. These plans include three full‐arc VMAT prostate plans, three left chest wall plans delivered using irregular compensators, two half‐arc VMAT lung plans, three MLC‐collimated static‐field pairs, and two SBRT liver plans. Finally, plans are reweighted to deliver the same number of monitor units, and mean dose to target volumes and organs at risk are calculated and compared.

It is important to note that this study is not intended to favor one vendor over another. Rather, the intent is to provide the reader with ample information so they can make an informed decision concerning the correct detector for the scanning of their system. Also, this study does not make any comparison to Monte Carlo simulations, since they would provide a “gold standard” for comparison, while the intent of this study is to compare actual scan data in actual clinical environments. Such comparisons are, however, valuable and available in the literature.^(14)^ Additionally, in an effort to reduce the number of variables influencing the ultimate outcome, comparisons of calculated fluence to actual fluence using matrix arrays, and their subsequent gamma analysis, are not used in this study.^15^, ^16^, ^17^, ^18^, ^19^

## II. MATERIALS AND METHODS

### A. Detectors

#### A.1 Ionization chamber

Advantages of ion chambers include their stable response across varying energy, dose, and dose rate, as well as the relatively low cost and high availability.^(2)^ The main disadvantage in its use is its finite size, which can result in a volume‐averaging effect. The ionization chamber used in this study is the CC13 (IBA Dosimetry GmbH, Schwarzenbruck, Germany), a vented, waterproof, and fully guarded ionization chamber designed for absolute and relative dosimetry. Its design allows for both axial and lateral beam entrance; however, only lateral beam entrance is used in this study. The outer and inner electrodes are both constructed from Shonka air‐equivalent plastic (C‐552) with density of $1.73\;g/{\text{cm}}^{3}$, while the chamber stem is constructed of PEEK (polyether ether ketone) with a density of $1.32\;g/{\text{cm}}^{3}$. The outer diameter of this chamber is 6.8 mm, the active length is 5.8 mm, and the nominal active volume is 0.13 cm^3^. Setup for the ionization chamber takes into account the effective point of measurement, adhering to the recommendations of AAPM's Task Group 51.^(20)^

#### A.2 Silicon diodes

The main advantages of silicon diode detectors are their fast response time, high spatial resolution, and high sensitivity.^(1)^ A disadvantage of the use of diodes is their dependence on dose rate and energy. Also, an inherent problem of using diodes is the overresponse to low‐energy scattered radiation, resulting from the relatively high photoelectric cross section of silicon (Z=14).^6^, ^21^ This overresponse can be as high as 10% for large fields.^(22)^ The overresponse is reduced by the use of metal shielding (such as in the case for the PTW 60008), which presents perturbation in measurement in the buildup region and small field sizes.

This study includes scanning using the PTW 60012 (Freiberg, Germany), an unshielded silicon diode detector which is housed in a polymer encasement. Unlike the other detectors used in this study, the PTW 60012 is aligned vertically, with its axial plane perpendicular to, and centered on, the central axis of the beam. Another solid‐state detector used in this study is the EDGE Detector model 1118 (Sun Nuclear Corporation, Melbourne, FL). The EDGE Detector is designed specifically for use in radiation beam scanning. This active detecting element in the EDGE Detector is 0.8 mm×0.8 mm, housed in brass, and is at a water‐equivalent depth of 0.5 mm from the top surface of the detector. As shown in Table 1, this detector has a comparatively high sensitivity, and results in lower signal noise or standard deviation of signal. When setting up this detector for profile measurements, the source‐to‐detector distance is the distance to the depth of the surface of the detector plus 0.5 mm for water equivalence. This is in spite of the fact that the detector's physical depth is 0.3 mm below the detector surface. It is worth noting that, though it is not a focus of this study, for percent depth dose measurements, one would align to the physical depth of 0.3 mm (not the 0.5 mm effective depth in water).

**Table 1:** Detectors used in the current study, along with their respective specifications

*Detector Type*	*Detector Model*	*Detector Manufacturer*	*Active Volume (cm* ^*3*^ *)*	*Detector Width (mm)*	*Cavity Radius (mm)*	*Shift (mm)*	*Sensitivity (nC/Gy)*	*Housing* / *Shell Material*	*Reference Detector*
Ionization Chamber	CC‐13	IBA	0.130	‐	3.0	−1.8	3.8	Shonka C552	CC‐13
Silicon Diode	60012	PTW	0.0025	1.12	‐	0.06	175	Polymer Plastic	60012
Silicon Diode	1118 EDGE Detector	Sun Nuclear	0.0019	0.8	‐	0.05	32	Brass	60012

### B. data acquisition

#### B.1 Accelerator

All data were acquired on a Varian TrueBeam (Version 1.6). This accelerator featured the Millennium 120‐leaf multileaf collimation system as a tertiary jaw. In the current study, the 6 MV flattened field was modeled. To ensure machine consistency between detector‐specific datasets, all data were acquired on the same day. Scans were performed using a nominal dose rate of 600 cGy/min at $D_{\text{max}}$.

#### B.2 Scanning equipment

**Figure 2:**
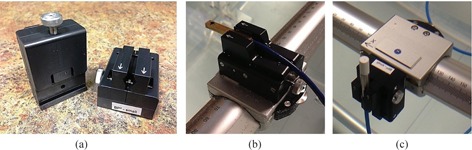
In the current study, all the detectors are setup horizontally, with the exception of the PTW 60012 diode: (a) the IBA universal detector mount allows stable and reproducible setup of detectors in the BluePhantom^2^, both horizontally and vertically; (b) the horizontal orientation is shown with the mounting of the EDGE Detector; (c) the vertical orientation is demonstrated with the mounting of the PTW 60012.

All data were acquired using a Blue Phantom^2^ three‐dimensional scanning system (IBA Dosimetry GmbH). This unit's universal detector mount allowed stable and consistent setup without perturbation of the initial setup. To ensure consistency of tank setup between detector‐specific measurements, all data were acquired during a single tank setup and alignment. The only differences between datasets were the detectors themselves. All detectors are attached to the scanning system using the IBA NP20‐100 universal detector holder which accepts detectors with diameters from 4 mm to 10 mm (shown in Fig. 2). Prior to each detector‐specific set of scans, the water level and isocenter were set in the scanning system. A central axis test was performed for each detector of verify positioning and orientation. Profile data from field sizes of 30 cm×30 cm and above were acquired in a shifted‐tank configuration. For these scans, asymmetric data are acquired with a greater lateral extent due to the alignment offset (in this case, 15 cm).

#### B.3 Common scan parameters

Scanning was performed in a slow, 0.3 cm/sec continuous method (with a 2 cm/sec nonmeasuring positioning speed). The continuous method is favored by the authors because the water is less disrupted as compared to the step‐by‐step motion, which can substantially change detector depth and source‐to‐surface distance. Following scanning, and prior to beam modeling, no smoothing or interpolation was performed on the data. The use of corrective smoothing algorithms presents an opportunity to increase penumbra sizes and reduce the natural horn effect that occurs at shallow depths. Data were acquired with 0.1 mm resolution.

### C. Beam modeling

Data acquired during scanning were formatted for, and subsequently imported into, Eclipse's beam configuration module. Three nonclinical beams were modeled for use by the Acuros XB algorithm — one model for each of the detectors used for profile scanning. This algorithm requires an assortment of relative scanned data, absolute point doses, and relative output factors in order to model the beam. The focus of this study is to show the clinical impact caused by the choice of detector with respect to its ability to accurately measure dose in the penumbra. Therefore, relative scanned profiles are the independent variable. Regardless of the detector being used for scanning profiles, certain required beam data remain essentially consistent across all studies.

For percent depth‐dose measurements, Varian recommends that acquisition should not be performed using mixed types of detectors (for example, ion chambers and diodes), for this might result in the erroneous calculation of the electron contamination model.^(23)^ In the current study, all PDD data were acquired using the CC13 chamber. Watts^(9)^ showed that, for both 6 MV and 18 MV photon beams, PDD values measured with the EDGE Detector and the CC13 agreed within 1% for a 10 cm×10 cm field. Output factors are measured using the daisy‐chained method which, for this case, hybridizes measurements using the EDGE Detector and the CC13.^(24)^ With the exception of beam profiles, the beam configuration is identical for each model.

Acuros XB is a volumetric dose calculation model available in the Eclipse treatment planning system. This algorithm provides the phantom scatter data for monitor unit calculations, which are based on measured output factors and absolute calibration factors. These monitor units are further tweaked with the inclusion of the effects of head and collimator scatter, which are included in the machine‐specific default source model and precalculated using Monte Carlo. Cross‐plane profiles were acquired at the beam's $d_{\text{max}}$ (1.4 cm), as well at depths of 5, 10, 20, and 30 cm. Profiles were measured for beams collimated with the secondary jaws at field sizes of 2 cm×2 cm,3 cm×3 cm,4 cm x×4 cm,6 cm×6 cm,8 cm×8 cm,10 cm×10 cm,12 cm×12 cm,15 cm×15 cm,20 cm×20 cm,30 cm×30 cm, and 40 cm×40 cm. These fields were chosen as recommended by the vendor for the modeling of the Acuros XB algorithm.

The Acuros model is considered a multiple‐source model,^(25)^ where dose to any voxel within the calculation volume represents the contribution from the primary source, the second source, and the electron contamination source. The primary source is represented by a point source located on the target plane along the central axis. The initial photon energy spectra along the CAX were modeled using the Monte Carlo transport code, BEAMnrc, based on realistic dimensions and material composition.^(26)^ The second source represents extra focal radiation, or all photons which originate from outside the Bremsstrahlung target, which include scatter contributions from the primary collimators, flattening filter, jaws, and MLCs. This finite virtual source is modeled using a two‐dimensional Gaussian, which is located just below the flattening filter. Electron contamination source represents the extra dose which is deposited in the buildup region (and not described by the primary and second source). This source provides the model with a depth‐dependent contamination dose curve for subsequent calculation.

In Eclipse, beams are modeled in an iterative optimization process which attempts to fit calculated model data to measured data (the former being calculated in an infinite slab of homogeneous water). The reader is encouraged to refer to the work of Tillikainen and colleagues^(27)^ for an in‐depth explanation of this optimization process. For the primary photon source, a curve is fitted, which gives the mean energy as a function of radial distance from the CAX. This curve is used to account for beam hardening from the flattening filter. The variation of photon fluence below the flattening filter is accounted for by fitting an intensity curve, which varies as a function of radial distance from the CAX.

The shape of the two‐dimensional Gaussian, which defines the second source, is adjusted so that calculated profiles match measured profiles. This includes adjustment of the width of the Gaussian at the (second) source plane, the relative intensity of the second source, and the mean energy of the second source.

During the optimization process, a two‐dimensional Gaussian curve is fit to match the measured data by adjusting the width of the second source. Following this fit, the relative intensity of the second source and the mean energy of the second source are empirically derived.^(28)^ The source width and Gaussian height parameters are attributed to penumbra shape and width.^(13)^

### D. Model and plan evaluation

The clinical plans used in this study were originally created and optimized using the beam model generated by CC13 measurements. Geometrically identical plans were created with the beam models, which were based on scan data from the other detectors. In order to generate comparable plans across all detector‐specific beam models, all fields of the additional models were weighted so that they delivered the same number of monitor units as the original CC13 plan. Thus, any dose difference‐to‐target‐volumes and organs at risk are isolated to differences in beam model.

#### D.1 VMAT prostate

**Figure 3:**
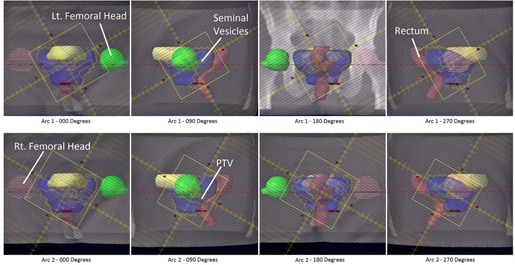
A plan was generated, consisting of two full arcs, which delivered 6840 cGy in 38 fractions to a patient's prostate bed for treatment of adenocarcinoma of the prostate following radical prostatectomy.

Two VMAT prostate plans were generated. The first plan consisted of two full arcs, which delivered 2340 cGy in 13 fractions as a boost to a prostate bed treatment. The first arc delivered 311 MU during a 360° clockwise arc, with the collimator rotated to 30°. The second arc delivered 419 MU during a counterclockwise traversal about the patient, with the collimator rotated to 330°. For each arc of this plan, a simulated beam's eye view (rotated to cardinal angles) is shown in Fig. 3, with PTV and organs at risk displayed. The second plan consisted of two full arcs, which delivered 7740 cGy in 43 fractions to treat an intact prostate. The first arc delivered 281 MU during a 360° clockwise arc, with the collimator rotated to 30°. The second arc delivered 379 MU during a counterclockwise traversal about the patient, with the collimator rotated to 330°.

#### D.2 Left chest wall using irregular compensators

**Figure 4:**
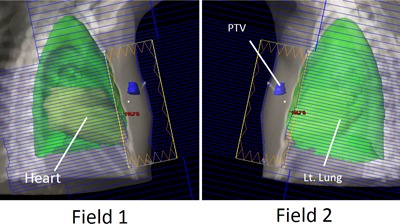
A plan was generated, consisting of two tagential fieds, designed to deliver 4256 cGy to the left chest wall in 16 fractions.

Three left chest wall plans were created, which used a pair of tangential fields delivering a conformal fluence where beam intensity was modulated using the MLCs as an irregular compensator. The first plan delivered 4256 cGy to the PTV in 16 fractions. The first field delivered 230 MU using a 300.4° gantry rotation with a 12° collimator rotation, while the second field delivered 230 MU using a 122.2° gantry rotation and a 348.0° collimator rotation. For each field of this plan, a simulated beam's eye view is shown in Fig. 4, with PTV and organs at risk shown. The second plan delivered 5000 cGy to the PTV in 25 fractions. The first field delivered 207 MU using a 303.0° gantry rotation with a 13° collimator rotation, while the second field delivered 180 MU using a 127.0° gantry rotations and a 347.0° collimator rotation. The third plan delivered 4256 cGy to the PTV in 16 fractions. The first field delivered 256 MU using a 303° gantry rotation with a 5° collimator rotation, while the second field delivered 226 MU using a 126° gantry rotation and a 355° collimator angle.

#### D.3 VMAT lung

**Figure 5:**
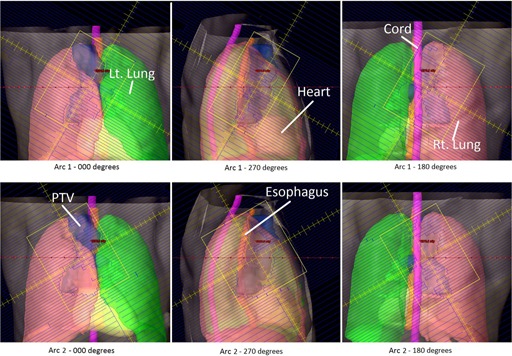
A VMAT plan was generated which delivered 6000 cGy in 30 fractions to a lesion in the patient's right lung resulting from small‐cell lung cancer.

Two VMAT lung plans were generated. In the first plan, a pair of half‐arcs delivered 6000 cGy in 30 fractions to a lesion in the patient's right lung. The first arc delivered 272 MU during a 180° clockwise arc, with the collimator rotated to 30°. The second arc delivered 302 MU during a 180° counterclockwise arc, with the collimator rotated to 330°. For each arc of this plan, a simulated beam's eye view (rotated to cardinal angles) is shown in Fig. 5, with PTV and organs at risk displayed. In the second plan, a pair of half‐arcs delivered 5000 cGy in 20 fractions to a lesion in the patient's left lung. The first arc delivered 316 MU during a 180° clockwise arc, with the collimator rotated to 30°. The second arc delivered 330 MU during a 180° counterclockwise arc, with the collimator rotated to 330°.

#### D.4 MLC‐collimated static field

**Figure 6:**
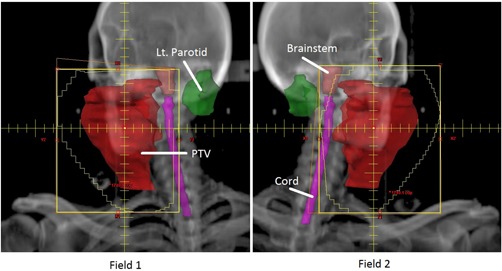
A wedged‐pair plan was generated to deliver 1400 cGy in 4 fractions to the PTV. The PTV encompassed a right neck mass (squamous cell carcinoma of the right tonsil) which measured greater than 6 cm, extending into the lateral nasopharynx, invading the pterygoid muscles, and skull based.

Three static‐field plans were all collimated with the MLCs and all used enhanced dynamic wedges. The first plan was a wedged‐pair designed to deliver 1400 cGy in 4 fractions to the PTV. The PTV encompassed a right neck mass (squamous cell carcinoma of the right tonsil) which measured greater than 6 cm. To create a more uniform dose through this narrow region, the fluence of each field was modified with an enhanced dynamic wedge. The first field delivered 244 MU from a gantry rotation of 17°, with a collimator rotation of 90° and with a 20° wedge angle. The second field delivered 256 MU from a gantry rotation of 197°, with a collimator rotation of 0° and with a 15° wedge angle. Both fields reduced organ‐at‐risk doses through MLC shaping. For each field of this plan, a simulated beam's eye view is shown in Fig. 6, with PTV and organs at risk shown. The second plan was a wedged‐pair plan designed to deliver 6300 cGy in 28 fractions to the PTV. The PTV encompassed a portion of the larynx. To create a more uniform dose through this narrow region, the fluence of each field was modified with an enhanced dynamic wedge. The first field delivered 148 MU from a gantry rotation of 270°, with a collimator rotation of 90° degrees and with a 30° wedge angle. The second field delivered 147 MU from a gantry rotation of 90°, with a collimator rotation of 90° and with a 30° wedge angle. The final plan was a three‐field plan designed to deliver 500 cGy in 10 fractions to the PTV. The PTV encompassed a portion of the spleen. To create a more uniform dose in the target region, the fluence of each field was modified with an enhanced dynamic wedge. The first field delivered 22 MU from a gantry rotation of 0°, with a collimator rotation of 0° and with a 30° wedge angle. The second field delivered 26 MU from a gantry rotation of 180°, with a collimator rotation of 90° and with a 30° wedge angle. The third, and open, field delivered 19 MU from a gantry rotation of 90° and a collimator rotation of 0°.

#### D.5 SBRT liver

**Figure 7:**
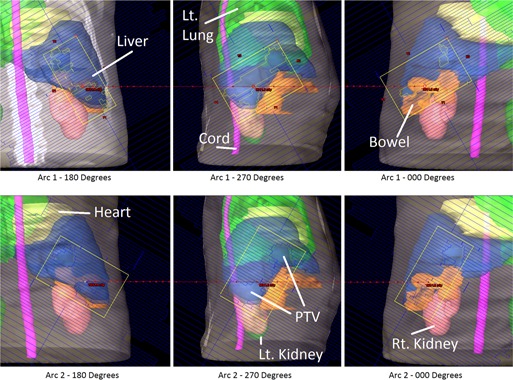
An SBRT plan was created to deliver 4800 cGy in 3 fractions to a pair of lesions in the patient's liver.

An SBRT plan was created to deliver 4800 cGy in 3 fractions to a pair of lesions in the patient's liver. This plan consisted of two 180° arcs. The first arc delivered 2252 MU during a clockwise rotation, with the collimator rotated to 30°. The second arc delivered 2491 MU during a counterclockwise rotation, with the collimator rotated to 330°. For each arc of this plan, a simulated beam's eye view (rotated to cardinal angles) is shown in Fig. 7, with PTV and organs at risk displayed. Another SBRT plan is also presented where 5400 cGy is delivered to a single lesion in a patient's liver. This plan consisted of two 180° arcs. The first arc delivered 3320 MU during a clockwise rotation, with the collimator rotated to 30°. The second arc delivered 3320 MU during a counterclockwise rotation, with the collimator rotated to 330°.

## III. RESULTS & DISCUSSION

### A. Scanning

The high‐Z silicon diode material of the PTW 60012 (and its respective higher cross section for photoelectric interaction at low energy) combined with the low energy scatter associated with larger field sizes by increased phantom scatter and collimator, result in a relative over‐response outside the primary beam. This effect is evident in penumbra measurements, as well as measurements of dose in the tails of the profiles.

**Figure 8:**
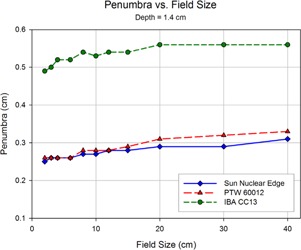
The penumbra for each detector as measured during the scanning procedure, and calculated as the lateral distance between the 80% and 20% values (normalized to the central axis). The relatively large size of the detecting volume of the ionization chamber results in volume averaging, yielding larger penumbra values. As the field size increases, greater lateral scatter results in increasing penumbral widths. This increase is minimized at the initial depth of 1.4 cm, and increases with depth.

**Figure 9:**
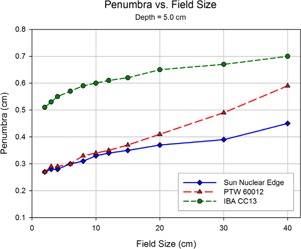
As scan depth increases, penumbra values for the diodes begin to converge with those of the ionization chamber. While the penumbra increases for all detectors as the distance from the source increases, scatter‐induced penumbral broadening affects mainly the diodes. The volume averaging of the detector suppresses the majority of this penumbral broadening. Also, the over‐response of the PTW 60012 begins demonstrating a profound effect at larger field sizes.

**Figure 10:**
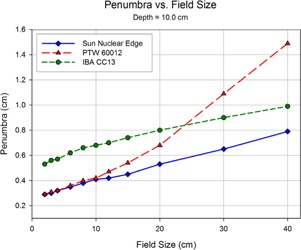
The overresponse of the 60012 becomes even more apparent as depth increases. The relatively high cross section for photoelectron production in silicon results in more photoelectron contamination in regions of increased low‐energy scatter. Since the 60012 is unshielded, this effect becomes pronounced.

**Figure 11:**
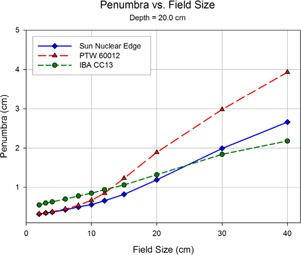
At a depth of 20 cm, the penumbral values of the CC13 and the EDGE detector begin to reach a near convergence as the scatter‐induced penumbral blurring reaches the magnitude of the volume averaging effect seen in the CC13 thimble ionization chamber.

**Figure 12:**
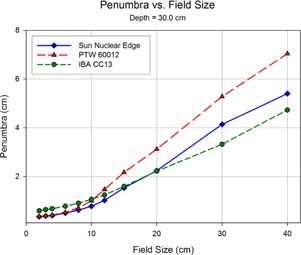
At the final depth scanning in the current study, 30 cm, the penumbral values of the CC13 and the EDGE show a clear separation from those measured by the 60012.

Shown in Figs. 8 to 12 are plots of the measured penumbra for each profile as a function of the nominal collimator width (as projected at isocenter). The penumbra is defined as the lateral distance between the 80% and 20% isodose lines as normalized to the dose at the central axis. At small field sized, both diode detectors yielded sharper penumbra than the larger‐volume ion chambers as a result of the volume averaging effect associated with finite‐size detectors. As phantom scatter increases with depth, and collimator scatter increase with field size, the 60012's higher response is noted.

**Figure 13:**
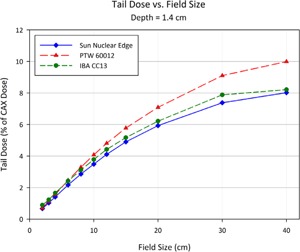
Plot of the dose to a reference point in the tail of the profile at a depth of 1.4 cm, the dose being represented as a percentage of the normalized dose to the central axis. The reference point for comparison is defined as 1.8 cm beyond the depth‐corrected geometric field width, as used for model evaluation by Rangel et al.^(13)^

**Figure 14:**
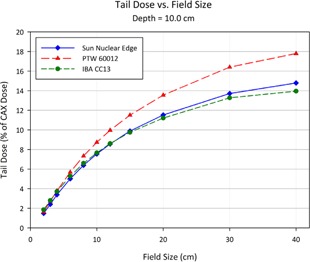
Plot of the dose to a reference point in the tail of the profile at a depth of 10.0 cm, the dose being represented as a percentage of the normalized dose to the central axis. The reference point for comparison is defined as 1.8 cm beyond the depth‐corrected geometric field width, as used for model evaluation by Rangel et al.^(13)^

**Figure 15:**
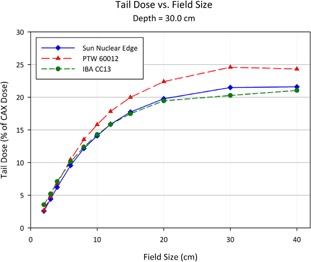
Plot of the dose to a reference point in the tail of the profile at a depth of 30.0 cm, the dose being represented as a percentage of the normalized dose to the central axis. The reference point for comparison is defined as 1.8 cm beyond the depth‐corrected geometric field width, as used for model evaluation by Rangel et al.^(13)^

Figures 13 to 15 show plots of the dose to a reference point in the tail of the profile, the dose being represented as a percentage of the normalized dose to the central axis. The reference point for comparison is defined as 1.8 cm beyond the depth‐corrected geometric field width, as used for model evaluation by Rangel et al.^(13)^ The PTW 60012 produced higher relative responses than the other detectors in tail, with the effect most pronounced at larger field sizes. The high‐Z silicon diode material of the PTW 60012 (and its respective higher cross section for photoelectric interaction at low energy) combined with the low energy scatter associated with larger field sizes by increased phantom scatter and collimator, result in an overresponse. As shown in Figs. 13 to 15, this overresponse increases with increasing field size.

As a means of quantifying the difference between the profiles measured during scanning and those calculated during the beam modeling process, a 1%/3 mm gamma error analysis is performed at 1 mm increments for each measured scan (Table 2). Individual gamma error values are averaged across all field sizes based on their region. There are three regions where test points are measured: inside the field (within the 80% isodose line), within the penumbra region (within the 80% and 20% isodose lines), and outside the field (below the 20% isodose line). There is a clear distinction between measured and calculated data between the models when looking in the region outside the field. The lower gamma error values calculated for the CC13 and EDGE models suggest greater confidence in model fidelity when comparing to the 60012 model.

**Table 2:** The average gamma error (1%, 3 mm) between measured profile data and profile data calculated by Eclipse during beam modeling

	*Average Gamma Error*		
*Test Point Location*	*CC13*	*60012*	*EDGE*
Inside Field (>80%)	0.20	0.20	0.20
Penumbra Region (<80% &>20%)	0.24	0.43	0.23
Outside Field (<20%)	0.36	0.92	0.32

### B. Planning

Tables 3 to 7 show the mean dose of the planning target volume (PTV), as well as relevant organs at risk (OAR) for each treatment plan, and each detector‐specific model. Mean doses to all PTVs and OARs agreed within 1% when comparing calculations using the CC13 model and the EDGE model. Since there is such a close agreement with the mean dose values calculated with the CC13 and EDGE, for subsequent discussion, mean dose values for volume computed using the 60012 model are compared to an average of the CC13 and EDGE model. Table 7 also includes the conformity index, which is a metric used to evaluate the tightness of a fit of a targeted volume to the isodose of the prescription.

Determining the relative intensity of the second‐source Gaussian is part of the modeling process, where dose in the tail region has an effect on subsequent calculations. The overresponse of 60012 in this region results in a larger relative intensity of the second source, and leads to an overestimation of scatter dose to regions which fall outside of the collimated target region. The relative intensities of the second source for the CC13 model and the EDGE model were nearly identical as a result of their similarity of relative dose in the tail region determined during scanning. When calculated with the 60012 model, all plans yield a higher mean dose to all OARs which spend some time out of the jaw‐collimated primary field. Figure 4 shows the beam's eye view (BEV) for the wedged field pair. The PTV remains central to the field, and the mean dose for the 60012 model is 0.1% lower than that of the average value from the CC13 and EDGE models. The brainstem remains mostly within the jaw‐collimated field; however, it is partially blocked by the MLCs. Since profile measurement differences only affect jaw collimation, MLC‐collimated regions remain unaffected by model differences. The mean dose to the brainstem is, however, slightly higher when calculated using the 60012 model a result of the superior aspect of the brainstem coinciding with the Y2 jaw. The overresponse of the detector in this region affects the model directly, resulting in higher scatter doses for structures beneath jaw edges and structures completely blocked by jaws. This trend continues in this example with respect to the mean dose to the cord. The cord has a large fraction of its volume outside of the jaw‐collimated field. Finally, the most substantial difference between the mean doses is that of the left parotid gland. The dose to this OAR almost exclusively results from scattered radiation, suggesting that the higher mean dose results in the 60012 model result from the higher readings in this region during the scanning process.

The finite size of the CC13 causes larger penumbras and a slightly larger field size, resulting in Eclipse calculating a smaller value of the width the second source. An example of this clinical manifestation is in the fact that plans calculated using the CC13 and the EDGE models showed a strong agreement, though these detectors yielded large differences in measured penumbra widths during scanning. These results agree with those of Rangel et al.,^(13)^ who showed that a 1 mm change in penumbral width results in a mere 0.01% change in horn height (as defined 1.8 cm within the geometric field edge) and a negligible effect everywhere else on the curve. Also, this clinical independence is partially the result of the fact that the measured penumbras are those at the jaw‐shaped field edge, not the MLC‐shaped field edge (with which all of these plans are shaped). The negligibility of penumbral differences agree with a recent Varian White Paper which suggested that the accurate measurement of the scanned profiles does not affect the configured model, but merely makes easier the direct comparison between the calculated and measured values.^(3)^

**Table 3:** Mean dose values for planning target volumes (PTV) and organs at risk (OAR) for the evaluated prostate plans, and for each of the detector‐specific models. The percent difference from the CC13 values are calculated by subtracting the tested values by the CC13 values, and normalized to 100% using the CC13 values

		*CC13*	*60012*	*% diff from CC13*	*EDGE*	*% diff from CC13*
*VMAT Prostate Plan 1*						
Mean Dose (cGy)	PTV	7204	7163	−0.6	7201	0.0
	Bladder ‐ CTV	4738	4719	−0.4	4736	0.0
	Seminal Vesicle	7159	7126	−0.5	7157	0.0
	Left Femoral Head	2077	2081	0.2	2076	0.0
	Right Femoral Head	2067	2072	0.2	2067	0.0
	Rectum	3828	3819	−0.2	3827	0.0
*VMAT Prostate Plan 2*						
Mean Dose (cGy)	PTV	7831	7796	−0.4	7829	0.0
	Bladder ‐ CTV	8229	8194	−0.4	8228	0.0
	Seminal Vesicle	7823	7789	−0.4	7821	0.0
	Left Femoral Head	1485	1487	0.1	1484	0.0
	Right Femoral Head	1524	1526	0.2	1524	0.0
	Rectum	3114	3112	−0.1	3114	0.0

**Table 4:** Mean dose values for planning target volumes (PTV) and organs at risk (OAR) for the evaluated left chest wall plans, and for each of the detector‐specific models. The percent difference from the CC13 values are calculated by subtracting the tested values by the CC13 values, and normalized to 100% using the CC13 values

		*CC13*	*60012*	*% diff from CC13*	*EDGE*	*% diff from CC13*
*Left Chest Wall Plan 1*						
Mean Dose (cGy)	PTV	4543	4538	−0.1	4543	0.0
	Left Lung	203	207	2.0	203	0.0
	Heart	115	121	5.4	115	−0.2
*Left Chest Wall Plan* 2						
Mean Dose (cGy)	PTV	5102	5096	−0.1	5100	0.0
	Left Lung	560	566	1.0	560	−0.1
	Heart	107	114	6.8	107	−0.5
*Left Chest Wall Plan* 3						
Mean Dose (cGy)	PTV	4466	4459	−0.2	4466	0.0
	Left Lung	490	494	0.9	490	−0.1
	Heart	98	105	7.6	99	−0.6

Using the 60012 beam model, in all plans, the target volume received an estimated dose which was lower than the dose calculated with the CC13 and EDGE models. This deviation was more pronounced with deep‐seated target volumes (such as in the lung, prostate, and spleen) and less pronounced with shallow volumes (such as in the larynx and chest wall).

**Figure 16:**
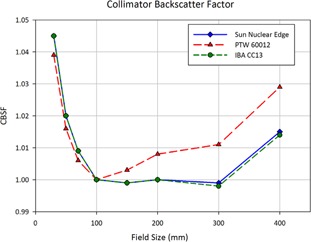
The collimator backscatter factor, or CBSF, is a derived quantity calculated by Eclipse in the last stage of beam modeling which serves as a conversion factor between measured output factors and those calculated using semi‐empirical models (based on scan data). In this plot, CBSF values are plot as a function square field size for each detector‐specific beam model used in the current study.

During beam modeling, Eclipse generates collimator backscatter factors, or CBSF, which are used in dose calculation for monitor unit calculation.^3^, ^29^ These are not to be confused with the in‐air output factor, or $S_{c}$, which characterizes the influence of collimator scatter for monitor unit calculation.^(30)^ The CBSF is a table of derived quantities used to fit measured total output factors to those calculated using the treatment planning system (modeled using scan data). The derivation of the CBSF values marks the final stage of modeling.^(28)^ Figure 16 shows the derived CBSF values for square field sized for each of the three models created during this study. A strong agreement is noted between the CBSF curves of the CC13 model and the Edge model. When comparing the three curves, one must note that a higher relative CBSF will result in a lower calculated monitor unit. When comparing two plans, if one calculates using the model with the higher CBSF and uses the higher number of monitor units (associated with the model with the lower value of CBSF), then in‐field structures will receive a higher dose. A noticeable difference is found between the CBSF curves of these models and that of the 60012 model. For fields larger than the reference field size (10 cm×10 cm), the difference between CBSF values increases with increasing field size (with the 60012 values being larger). When this model is used for monitor unit calculations, the effect is an increased dose to the noncollimated (target) region for fields larger than 10 cm×10 cm. For field sizes which are close to the reference field size, this difference is minimized. This is evident in plan comparisons using large field sizes, including left chest wall plans and static head and neck cases. The CBSF curves for field smaller than the reference field size again show a difference between the 60012 model and the models for the CC13 and EDGE Detector, with CBSF values being lower for the 60012. This difference becomes more pronounced with decreasing field size. Clinically, this effect results in a dose reduction to the target region. This is evident in prostate and lung plans, where the effective field sizes are much smaller than the reference field sizes.

**Table 5:** Mean dose values for planning target volumes (PTV) and organs at risk (OAR) for the evaluated lung plans, and for each of the detector‐specific models. The percent difference from the CC13 values are calculated by subtracting the tested values by the CC13 values, and normalized to 100% using the CC13 values

		*CC13*	*60012*	*% diff from CC13*	*EDGE*	*% diff from CC13*
*Lung Plan 1*						
Mean Dose (cGy)	PTV	6322	6312	−0.2	6327	0.1
	Lt. Lung	955	956	0.1	956	0.1
	Rt. Lung	2135	2141	0.3	2136	0.1
	Cord	1055	1061	0.6	1056	0.1
	Heart	265	273	3.3	265	0.2
*Lung Plan 2*						
Mean Dose (cGy)	PTV	5290	5274	−0.3	5288	0.0
	Lt. Lung	2590	2590	0.0	2589	0.0
	Rt. Lung	793	793	0.0	793	0.0
	Cord	736	738	0.3	735	0.0
	Heart	847	852	0.5	847	0.0

**Table 6:** Mean dose values for planning target volumes (PTV) and organs at risk (OAR) for the static‐field plans, and for each of the detector‐specific models. The percent difference from the CC13 values are calculated by subtracting the tested values by the CC13 values, and normalized to 100% using the CC13 values

		*CC13*	*60012*	*% diff from CC13*	*EDGE*	*% diff from CC13*
*Wedged Pair Plan*						
Mean Dose (cGy)	PTV	1447	1445	−0.1	1447	0.0
	Brain Stem	630	630	0.2	630	0.0
	Cord	734	735	0.2	733	0.0
	Lt. Parotid	60	64	0.3	60	0.0
*AP/PA Plan 1*						
Mean Dose (cGy)	Larynx (PTV)	6529	6530	0.0	6530	0.0
	Cord	67	71	7.2	67	−0.2
*AP/PA Plan 2*						
Mean Dose (cGy)	Spleen (PTV)	517	516	−0.1	517	0.6
	Cord	350	350	0.2	350	0.7
	Lt. Kidney	503	504	0.2	503	1.2
	Rt. Kidney	117	117	0.3	117	0.4

**Table 7:** Mean dose values for planning target volumes (PTV) and organs at risk (OAR) for the evaluated SBRT liver plans, and for each of the detector‐specific models. The percent difference from the CC13 values are calculated by subtracting the tested values by the CC13 values, and normalized to 100% using the CC13 values

		*CC13*	*60012*	*% diff from CC13*	*EDGE*	*CC13 % diff from*
*SBRT Liver 1*						
Mean Dose (cGy)	PTV	5216	5197	−0.4	5215	0.0
	Liver	1513	1513	0.0	1513	0.0
	Cord	231	231	0.1	231	0.0
	Rt. Kidney	1230	1231	0.1	1229	0.0
	Lt. Kidney	122	122	0.3	122	0.0
	Lt. Lung	68	68	0.7	68	0.0
	Bowel	692	697	0.7	692	0.0
	Heart	88	92	4.2	88	0.1
	Conformity Index	1.21	1.20	—	1.21	—
*SBRT Liver 2*						
Mean Dose (cGy)	PTV	6311	6301	−0.2	6311	0.0
	Liver	1582	1583	0.1	1581	0.0
	Cord	226	227	0.4	226	0.0
	Rt. Kidney	28	30	4.6	28	0.0
	Lt. Kidney	22	23	2.3	22	0.0
	Rt. Lung	83	84	0.4	83	0.0
	Lt. Lung	429	432	0.6	429	−0.1
	Heart	242	273	0.5	272	0.0
	Conformity Index	1.00	0.99	—	1.00	—

## IV. CONCLUSIONS

This study shows that for modeling the 6 MV beam of Acuros XB in Eclipse Version 11, the choice to use a CC13 scanning ion chamber or an EDGE Detector was an unimportant choice, providing nearly identical models in the treatment planning system. Penumbra width did not play a role. Though continually measuring larger values for penumbral widths than those of the EDGE Detector, the beam models for both detectors were very similar. Dose in the tail region of the profile made the largest difference. By overresponding in the tail region of the profile, the 60012 diode detector scan data affected the beam model in such a way that target doses were reduced by as much as 0.4% (in comparison to CC13 and EDGE data). This overresponse also resulted in an overestimation of dose to peripheral critical structure, whose dose contributed mainly of scatter. The term “overestimate” assumes the correct estimate of dose using beam models based on CC13 and EDGE Detector data. It is important to note that PTW does not recommend using the 60012 for such measurements, much for the same reasons mentioned in this study. This is, however, included to demonstrate the clinical effects from using a detector that is known to provide an overresponse in the tail region.

The authors would like to reiterate the fact this study compares these detectors for the 6 MV beam of a TrueBeam in Acuros XB of Eclipse V11. The reader is cautioned not to extrapolate these data for other energies, accelerators, detectors, algorithms, and treatment planning systems with a study similar to that presented herein.

## Supporting information

Supplementary Material
